# Aggressive low grade middle ear adenocarcinoma with multiple recurrences: a case report

**DOI:** 10.1186/1746-1596-6-62

**Published:** 2011-07-07

**Authors:** Nadia G Elhefnawy

**Affiliations:** 1 Electron Microscopy Unit of Ain Shams University Specialized Hospital, Pathology Department, Faculty of Medicine Ain Shams University, Cairo Egypt

**Keywords:** adenocarcinoma, aggressive, middle ear

## Abstract

**Background:**

Primary tumours of the middle ear are much less commonly encountered in clinical practice than non neoplastic lesions. Middle ear adenocarcinoma is a very rare, locally invasive neoplasm assumed to arise from the middle ear mucosa. Because the natural course and clinical behavior of this neoplasm are far from established, the sporadic reports of such cases continue to provide basis for better understanding.

**Case description:**

A case of low grade adenocarcinoma of the middle ear is described in details with regard to its clinical presentation, radiological findings, histopathological, immunohistochemical and ultrastructural findings. The tumour recurred four times.

## Introduction

As a consequence of their rarity, middle ear neoplasms may be difficult to classify, for both clinicians and pathologists (Devaney etal.2003) [1]. Middle ear adenocarcinoma is a very rare, locally invasive neoplasm assumed to arise from the middle ear mucosa. Although endolymphatic sac tumor (aggressive papillary middle ear tumor) and jugulotympanic paraganglioma may show brain invasion, intracranial extension of histologically confirmed middle ear adenocarcinoma has not been previously reported (Paulus etal 199) [2].

## Case Report

A male patient presented at age 24 with a mass in the right middle ear extending to the external auditory meatus. He underwent surgery and the mass was diagnosed as a simple polyp. One year later he presented with a recurrent bleeding mass. The mass was re-excised and the surgeon informed him that it was adherent to the facial nerve and the patient ended up with a facial paralysis. The mass was diagnosed as a middle ear adenoma. The patient was free of symptoms for 5 years when he presented with a recurrent reddish mass in the previous mastoid cavity filling it completely and peeping through the Eustachian tube. The mass was completely excised in September 2000 and the biopsy revealed a middle ear adenoma.

The patient was followed up and was free until 2003 when routine MRI showed a huge mass extending behind the mastoid cavity into the posterior cranial fossa. The previous mastoid cavity was free of recurrence. A posterior fossa craniotomy was performed and the mass partially excised as it was extremely bloody and adherent to the cerebellum and brainstem. The biopsy revealed it to be a middle ear adenocarcinoma. The patient received post-operative radiotherapy on the remnant and repeat follow-up MRI revealed it to be stable. In 2005 a follow-up MRI was ordered for recurrent headaches and revealed a large mass in the middle cranial fossa above the tegmen. The previous middle ear and mastoid cavities were still free of recurrence. The mass was excised by a middle fossa craniotomy and the last biopsy revealed an adenocarcinoma as well. The patient has no family history of similar condition

**Figure 1:**
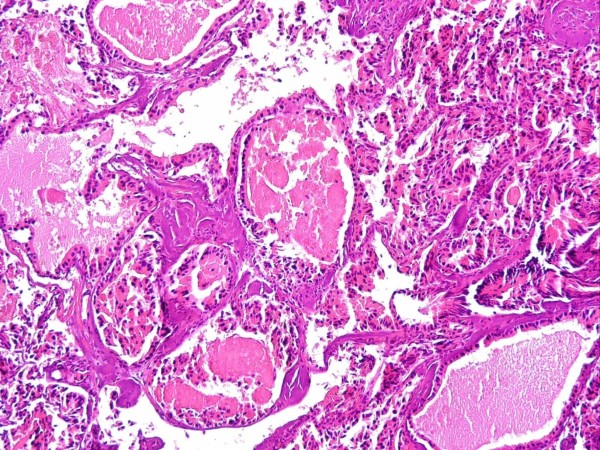
**A photomicrograph of the tumor**. It is made up of epithelial cells with eosinophilic cytoplasm, arranged in trabeculae and glandular spaces containing eosinphilic material. (H& E stain)

Histologically the tumour cells are arranged in islands, cords or form glandular spaces. The latter contain brightly eosinophilic secretory material. Tumour cells are cuboidal or oval with finely granular eosinophilic cytoplasm and centrally placed cytologically bland nuclei. (Figure 1)

**Figure 2:**
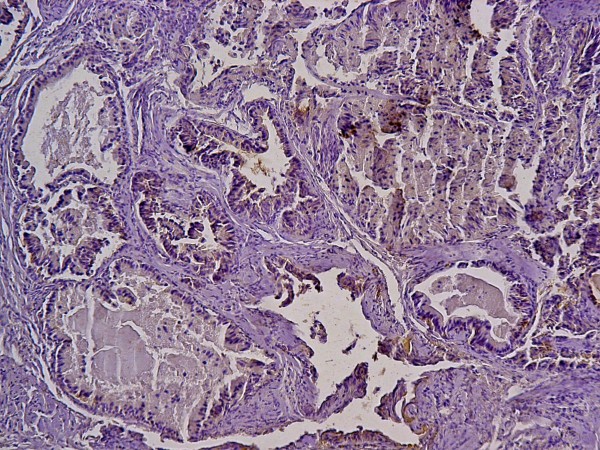
**positive immunohistochemical staining of the tumor cells for cytokeratin**.

**Figure 3:**
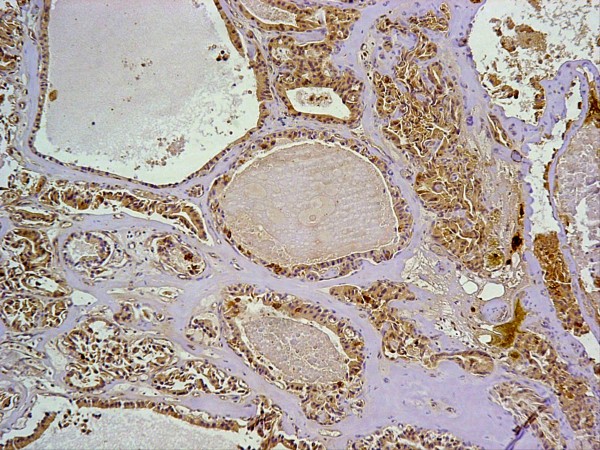
**Positive immunohistochemical staining of the tumor for vimentin**.

**Figure 4:**
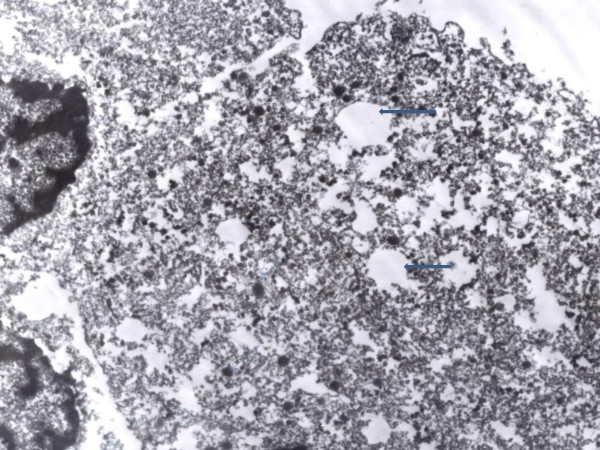
**An electron micrograph of the tumor cells showing apical vesicles (thick blue arrows) and electron dense granules (thin arrows)**. (original magnification 6000, urinyl acetate and lead citrate)

**Figure 5:**
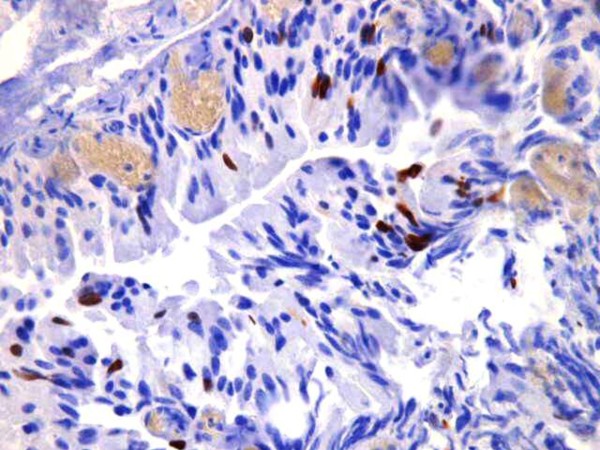
**Ki-67 immunostaining showing low proliferation index**.

Immunohistochemistry revealed that the tumour cells are positive for keratin (Figure 2), vimentin (Figure 3), and S100 protein but negative for chromogranin. Electron microscopy revealed evidence of exocrine differentiation in the form of apical membrane bound vesicles and electron dense granules. Surface microvilli have also been found (Figure 4). The proliferation index was assessed by Ki 67 immunostaining and it was about 1%. (Figure 5).

## Discussion

As epithelial tumors as a group are rarities in the middle ear, epithelial tumours of the middle ear of variable degrees of aggressiveness have been considered together in several reports [3-5]. Moreover an entity designated aggressive papillary middle ear tumour has been recognized; this term describes a middle ear tumour which may, in contrast to middle ear adenoma, act in a particularly aggressive fashion with intracranial extension [6,7]. Clearly, for the term middle ear adenoma to have any meaning in isolation, it must refer to a tumor which does not exhibit either light microscopic or clinical/radiologic evidence of aggressive or malignant behavior [1]. In this case report the light microscopic, immunohistochemical and electron microscopic findings were similar to those of middle ear adenoma. This tumor is considered to be low grade based on histology and low proliferation index but malignant based on aggressively invasive behavior, as it recurred four times and it was invasive to the petrous bone, posterior cranial fossa and middle cranial fossa.

The differential diagnosis of this tumour include papillary adenocarcinoma (aggressive papillary tumor) of the middle ear which has a papillary-to-glandular appearance and may produce a colloid-like substance and resemble papillary carcinomas of the thyroid. It has been suggested that this may be the same tumor as low grade adenocarcinoma of endolymphatic sac origin, which has locally extended into the middle ear. These tumors are immunohistochemically positive for cytokeratin, epithelial membrane antigen, and S100 protein. In the present case the tumour showed no papillae, and it remains to be verified whether similar tumours can originate from the middle ear. Paulus etal. 1999 [2] described a 53-year-old man who suffered from otalgia and tinnitus for more than 10 years and from neurological deficits for 1 year due to a large temporal bone tumor that invaded the temporal lobe. A combined neurosurgical and otolaryngological resection was performed. Pathological analysis revealed a low-grade adenocarcinoma of a mixed epithelial--neuroendocrine phenotype, which showed a close histological similarity to, and topographical relationship with, middle ear epithelium. The authors conclude that middle ear adenocarcinoma belongs to the spectrum of extracranial tumors that have possible local extension to the brain.

## Conclusion

Every individual case of middle ear adenomatous tumor requires a detailed clinical and radiological workup. All cases should be managed aggressively with an attempt at tumor removal. Regular follow-up with sequential radiology is essential.

## Consent

Written informed consent was obtained from the patient for publication of this case report and accompanying images. A copy of written consent is available for review by the Editor- in- Chief of this journal.

## Competing interests

The author declares that they have no competing interests.

## Authors' contributions

The author is responsible for data collection, and interpretation, literature search and manuscript preparation. She read and approved the final manuscript.
